# Microbial community structure in recovering forests of Mount St. Helens

**DOI:** 10.3389/frmbi.2024.1399416

**Published:** 2024-11-04

**Authors:** Mia Rose Maltz, Michael F. Allen, Michala L. Phillips, Rebecca R. Hernandez, Hannah B. Shulman, Linton Freund, Lela V. Andrews, Jon K. Botthoff, Emma L. Aronson

**Affiliations:** ^1^ Plant Science and Landscape Architecture, University of Connecticut, Storrs, CT, United States; ^2^ Center for Conservation Biology, University of California, Riverside, Riverside, CA, United States; ^3^ Microbiology and Plant Pathology, University of California, Riverside, CA, United States; ^4^ Department of Botany and Plant Sciences, University of California, Riverside, Riverside, CA, United States; ^5^ Land, Air, and Water, Resources Department, University of California, Davis, Davis, CA, United States; ^6^ Ecology and Evolutionary Biology, University of Tennessee, Knoxville, TN, United States; ^7^ Genetics, Genomics, and Bioinformatics Program, University of California, Riverside, Riverside, CA, United States; ^8^ Tecan Genomics, Redwood City, CA, United States

**Keywords:** succession, bacteria, fungi, AM fungi, Mount St. Helens, community assembly, legacy effects, diversity

## Abstract

**Introduction:**

The 1980 eruption of Mount St. Helens had devastating effects above and belowground in forested montane ecosystems, including the burial and destruction of soil microbes. Soil microbial propagules and legacies in recovering ecosystems are important for determining post-disturbance successional trajectories. Soil microorganisms regulate nutrient cycling, interact with many other organisms, and therefore may support successional pathways and complementary ecosystem functions, even in harsh conditions. Historic forest management methods, such as old-growth and clearcut regimes, and locations of historic short-term gopher enclosures (*Thomomys talpoides*), to evaluate community response to forest management practices and to examine vectors for dispersing microbial consortia to the surface of the volcanic landscape. These biotic interactions may have primed ecological succession in the volcanic landscape, specifically Bear Meadow and the Pumice Plain, by creating microsite conditions conducive to primary succession and plant establishment.

**Methods and results:**

Using molecular techniques, we examined bacterial, fungal, and AMF communities to determine how these variables affected microbial communities and soil properties. We found that bacterial/archaeal 16S, fungal ITS2, and AMF SSU community composition varied among forestry practices and across sites with long-term lupine plots and gopher enclosures. The findings also related to detected differences in C and N concentrations and ratios in soil from our study sites. Fungal communities from previously clearcut locations were less diverse than in gopher plots within the Pumice Plain. Yet, clearcut meadows harbored fewer ancestral AM fungal taxa than were found within the old-growth forest.

**Discussion:**

By investigating both forestry practices and mammals in microbial dispersal, we evaluated how these interactions may have promoted revegetation and ecological succession within the Pumice Plains of Mount St. Helens. In addition to providing evidence about how dispersal vectors and forest structure influence post-eruption soil microbiomes, this project also informs research and management communities about belowground processes and microbial functional traits in facilitating succession and ecosystem function.

## 1 Introduction

Natural disasters such as volcanic eruptions can potentially change change Earth’s ecological systems. Volcanic ash, lava, and debris flows have transformative and ecosystem-level effects, such as burying vegetation and destroying habitat. Volcanic eruptions may also dramatically affect atmospheric conditions and precipitation, far from where a blast originated, with extensive implications for both natural landscapes and human systems, such as in agriculture, industry, art, and religion (Stothers, 1984). Patchy mosaics of new soils emerge after volcanic eruptions, yielding shifts in substrate age, soil development, and primary successional processes (Vitousek, 2004). As volcanic eruptions resurface landscapes, the effects of the blast reverberate belowground as soil microorganisms may perish, aggregate within unaltered areas (as refugia), or disassociate with their hosts (Jackson et al., 2022). Belowground ecosystem functions likely diminish following eruptions, which may inhibit microbial activity and reestablishment (Hernández Garcia et al., 2020).

Thus, eruptions indirectly hinder plant reestablishment, nutrient cycling, and both provision and regulation of ecosystem services (Banwart et al., 2019). In these novel environments, microorganisms struggle to reassemble beneath the ashfall and pyroclastic flow (Dale et al., 2005).

The U.S. State of Washington is home to Mount St. Helens, a volcano known for numerous historic eruptions. During volcanic eruptions, fragmented material known as tephra is produced, ejected, and then deposited within the blast zone (Dale et al., 2005). At Mount St. Helens, tephra consisting of smaller particles (< 4 mm ash) to larger blocks (> 32 mm angular stones) is intercalated by buried forest floor layers in profiles that vary in organic matter content and nutrient concentrations (Allen et al., 2005). Mount St. Helens’s cataclysmic explosion in May 1980 destroyed more than 350 km^2^ of coniferous forest and montane habitats in the Cascades Mountains of the western United States, influencing not only aboveground vegetation and habitat for wildlife, but also free-living and host-associated soil microbial communities. The vertical stratification of soil horizons provides different resources for newly developing plant roots and rhizosphere microorganisms (Phillips and Lorz, 2008). Epidedons, subsurface edaphic layers, and soil aggregates engender spatial arrangements for roots or microbes to obtain limited resources, locate refugia, or proliferate amid the regolith. Pyroclastic flows created new environments from the crater to the Pumice Plain as remnant rubble from the May 1980 eruption from the crater to the Pumice Plain, new environments were created by pyroclastic flows as remnant rubble from the May 1980 eruption. These environments contained buried organic material and layers of tephra, with much of the blastfall zone covered in thick layers of snowpack, ash, and fallout from the blast (Findley, 1981).

Volcanic soils in forested ecosystems are exposed to high—often transformative—temperatures, as soil carbon (C) and nitrogen (N) transformation rates shift, leading to less N turnover, more N immobilization, and greater increases in C storage than are found in nonvolcanic soils (Yokobe et al., 2020). Post-eruption soils also tend to be more acidic, with more gas emissions (i.e., methane, CH_4_; carbon dioxide, CO_2_; hydrogen sulfide, H_2_S; hydrogen; H_2_ gases), giving rise to novel microbial communities with varied nutritional modes, such as those that are chemolithoautotrophic (Picone et al., 2020).

Although many lifeforms perished as the eruption transformed or translocated sterile tephra, some remnant soil organisms survived (Allen et al., 1984, 2018). Besides those buried under the ashfall, immigrant or ruderal taxa may have sought out shelter and found refugia in stark environments and the Pumice Plain’s sterile tephra, which initially consisted of pumice emerging from the pyroclastic flow. The pumice ranged from 10 – 20 mm in diameter and contained no measurable carbon (C) or nitrogen (N) (Fruchter et al., 1980; Baross et al., 1982). In 1981, scattered individuals of *Lupinus lepidus* (i.e., lupine) managed to establish patches (Allen and MacMahon, 1988; Allen et al., 1992, 2005) and likely associated with ruderal microorganisms, such as endosymbiotic rhizobial bacteria that are capable of atmospheric nitrogen fixation within the rhizosphere (Vuong et al., 2017; Coba de la Pena et al., 2018).

Lupine also served another important role in successional dynamics: as a prime food source for pocket gophers (*Thomomys talpoides*, Richardson), thus, inviting these ecosystem engineers (Reichman and Seabloom, 2002) to seed the Pumice Plain with biotic propagules. Gophers are known as “fossorial species,” meaning “hole diggers.” Findings from a fundamental study recounted by Logan (2007) showed that a single gopher can move 227 kg of soil per month, with gopher populations translocating 38,000 kg of soil per acre per year. Given that the disturbance intensity at Mount St. Helens differentially impacted pocket gophers, these parameters affected successional processes within both patchy mosaics and disturbance intensity gradients (Crisafulli et al., 2005; Dale et al., 2005). These patches subsequently metastasized as biotic communities grew proximally and distally (Franklin, 2005; Dale et al., 2005) within the blastfall zone. While the type, abundance, and distribution of biotic and abiotic legacies corresponded with this complex post-eruption landscape, a confluence of chemical, soil, and extant—or dead—biotic legacies may obfuscate patterns arising from the eruption, endogenous inputs, or effects of distant propagule sources.

At Mount St. Helens, the time required for landscapes to recover varied across systems and by the pre-eruption conductions. Spatial heterogeneity, responses to disturbance, and widespread biotic dispersal influenced the speed and variability of terrestrial and aquatic systemic response trajectories. After the 1980 eruption, lakes and streams became enriched with nutrients and resumed pre-eruption productivity levels far sooner than terrestrial ecosystems, as forest floors were blanketed by nutrient-poor tephra in response to dominant tree communities being extinguished. Geophysical forces, wildlife, and human activities contributed to the multifaceted dynamics during succession, including facilitation, inhibition, tolerance, and relay succession, with communities changing deterministically from pioneer to climax taxa (McCook, 1994; Pulsford et al., 2016). Yet researchers at Mount St. Helens also observed contemporaneous occurrences of early-seral and late-seral species, herbivory, predation, and mutualisms. Furthermore, ruderal and established taxa altered environmental parameters, such as by changing C and N concentrations to emulate more livable habitats (Halvorson et al., 2005).

In the clearcut, the ashfall covered the intact meadow soil to a depth of 12 cm. Because a deep snowpack was still in place in May, soil organisms, such as the pocket gopher, survived the eruption (Allen et al., 1984, 2018). The gophers burrowed back to the surface in spring, bringing old soil, reducing bulk density, and mixing in different soil layers and “stored topsoil”, with the ash mixture at approximately 1:1 soil to ash (Allen et al., 1984). These “islands” of inoculated soil included a diverse soil biota, mycorrhizal fungi, and nutrients (Allen et al., 2005). In 1980 (MacMahon and Warner, 1984), the organic C of the tephra was 2.2g.kg^-1^ and nitrate N 0.9mg.kg^-1^. However, the buried soil was highly organic (C was 39.6g.kg^-1^ with NO_3_-N of 10.9mg.kg^-1^). By 2000 (Allen et al., 2005), the organic C and N in the tephra remained low (1.46g.kg^-1^ and 10mg.kg^-1^, respectively), whereas the buried soil C averaged 17.5g.kg^-1^ and N averaged 390mg.kg^-1^). By 2015, the forb and shrub vegetation were dense.

**Figure 1:**
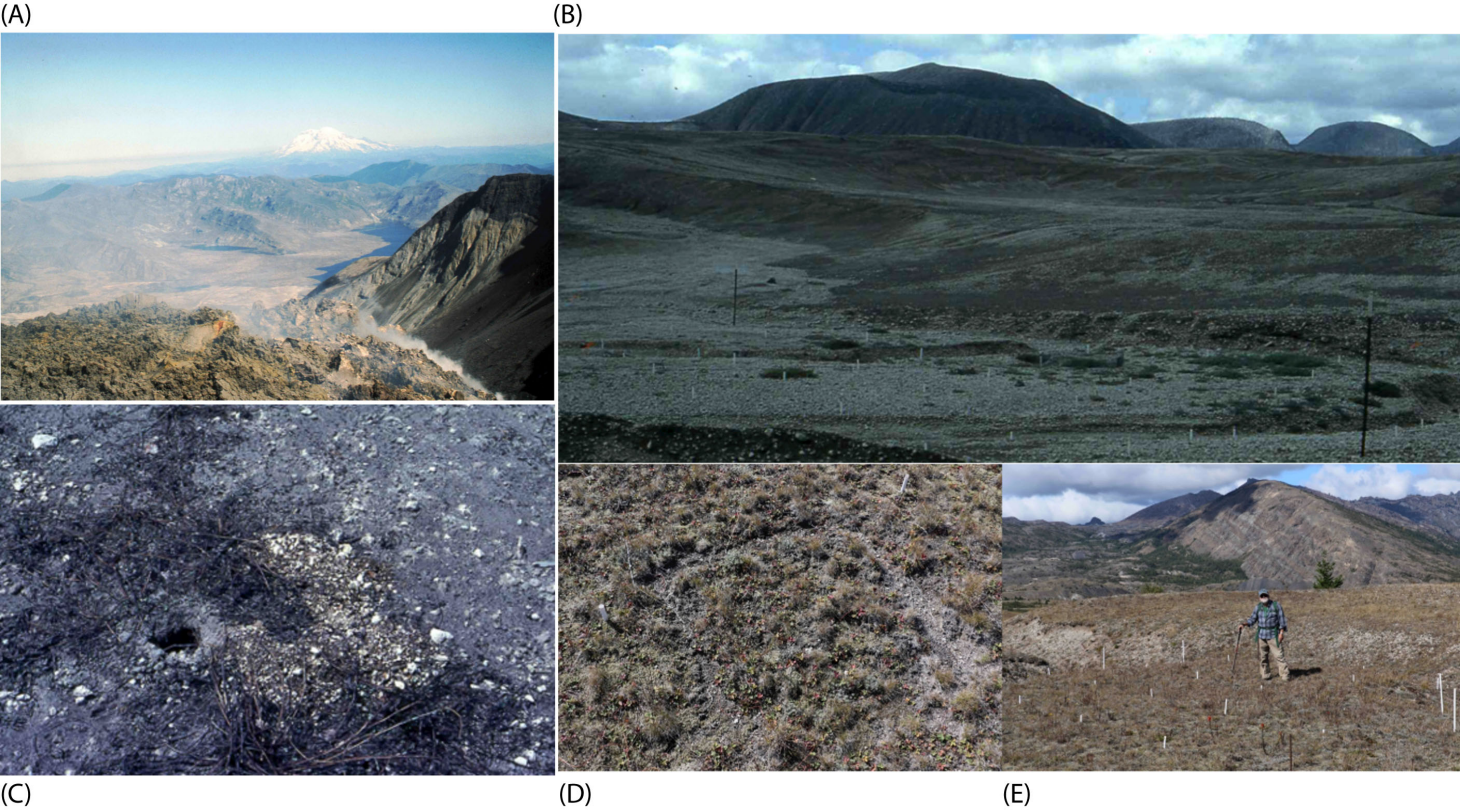
Overview of the Mount St. Helens study area. Aerial photograph above the dome in the crater, taken from a helicopter, above Mount St. Helens in 1982 **(A)**. Ashfall extends over Bear Meadow, with the Pumice Plain in the foreground and Mount Rainier in the background. (photograph taken by M.F. Allen). Pumice Plain from 1983 plots shown in **(B)**. **(C)** shows gopher activity and residual ash with old soil, transported by gophers in the Bear Meadow clearcut site. Photographic documentation shows an overview of the vegetation plots **(D)** in 1982 in the Pumice Plain, which corresponded to the year that gopher enclosures were set up following the pyroclastic flow, amidst the residual ash and tephra. Recent site visit in 2018 to historic gopher enclosure plots (M.F. Allen, shown in the foreground) within the Pumice Plain **(E)**.

The Pumice Plain site was within the pyroclastic flow consisting of sterile tephra, greater than 20m deep, upslope from Spirit Lake (**Figure 1**). Initially, the predominant feature was the texture of the growth medium, composed of marble- to golf ball-sized stones of pumice, with no measurable C or N (Fruchter et al., 1980; Baross et al., 1982). A few widely scattered individuals of *Lupinus lepidus* Dougl. had established during 1981. In 1982, these had reproduced, forming small patches. One of these became the focus of our research (Allen and MacMahon, 1988; Allen et al., 1992, 2005, 2018). In 1982, an individual pocket gopher (*Thomomys talpoides* [Richardson]) from a nearby clearcut was placed in a 1m^2^ enclosure around a single *L. lepidus* individual for 24 hours (Allen and MacMahon, 1988). By 1987, in the interspace total C was only 590 mg.kg^-1^ and N 17 mg.kg^-1^ (Halvorson et al., 2005). Under the plant, though, soil was already forming, and total C was 840mg.kg^-1^ and total N53mg.kg^-1^. By the year 2000, total C was 1.28 g.kg^-1^, and total N 114 mg.kg^-1^ in the interspace and C had increased to 4.6 g.kg^-1^ and total N to 384 mg.kg^-1^. Other characteristics can be found in Halvorson and colleagues (Halvorson et al., 2005). By 2015, the individual *L. lepidus* was long dead and gone, but a diverse suite of plants occupied the Pumice Plain extending up the mountain. Gophers had migrated back onto and up the Pumice Plain (Allen et al., 2018).

This seminal backdrop for studying secondary succession, in part due to the magnitude of the 1980 event, conveys valuable biological information to contextualize meaningful ecological issues, commensurate with thorough, protracted, and robust detection and monitoring of both the *types* and *levels* of remnant biological legacies herein (Franklin; Dale et al., 2005). As forest practices or gopher enclosures (as per Allen and MacMahon, 1988) overlay manipulative or experimental approaches, we are more equipped to disentangle the rules governing community assembly in this system subjected to natural disasters. The recent advent of cutting-edge technologies may facilitate fine-scale investigations of how top-down (e.g., clearcuts) or bottom-up (e.g., discrete or assisted microbial migration) factors influence long-term pedological trajectories and biotic succession of vegetation and macrofauna. Next-generation tools (Zimmerman et al., 2014), such as -omics approaches (Quince et al., 2017) and targeted-amplicon sequencing of fungal or bacterial communities (Taylor et al., 2016), may now be leveraged to track the arrival—and subsequent encroachment—of free-living, mutualistic, or commensal microorganisms in substantially sterile, harsh environments (Allen et al., 1984). In the wake of this historic blast, microbial communities—or their assisted migration—may have played a role in altering long-term successional processes and community assembly (Allen and MacMahon, 1988; Allen et al., 1992). Using next-generation tools, we can evaluate how, or to what extent, these effects may have lingered or attenuated over decadal scales since eruption events.

Human activities that altered landscapes, such as conventional forestry practices or modifying of fish and game species, may have influenced rates and patterns of ecological responses to the eruption. The spread of nonnative species, changes in primary production, and vegetation type conversion may exert selective pressures on soil microbial communities and likewise affect food webs (including birds or invertebrates), biodiversity, and ecosystem multifunctionality (Wagg et al., 2014). Dove and Keeton (2015) suggest that forest management practices directly influence microhabitat characteristics, such as those critical to forest fungi’s survival, which could indirectly affect decomposition and nutrient cycling. Forest structural complexity and functional diversity likewise affect these key soil processes. According to Grime’s (1998) mass-ratio hypothesis, changes to dominant plant species, and rates of primary production, such as by clearcut forestry management practices, could feed back to affect soil C and biogeochemical cycling, which may have implications for habitat quality, stability, and resilience to disturbance.

This study examined the effects of pre-eruption forest management and post-eruption gopher additions on bacterial and fungal communities, including plant-root-associated AM fungal mutualists. We hypothesized that there would be greater fungal and bacterial richness in old-growth forests after the blast than in previously clear-cut sites, e.g., Bear Meadow. Taxa originating in old-growth forests may be more resilient to disturbance or natural disasters than those altered by clearcutting forest practices (Bowd et al., 2022; Delgado-Baquierzo et al., 2018). In areas laden with volcanic debris, we predicted that old-growth and clearcut forest management processes may have opposing effects on fungal and bacterial groups over decadal time scales and correspond to changes in C:N ratio in soils. These conjectural effects may be related to the relative abundance of particular microbial groups, the functions performed by microbes, or litter quality from the surrounding vegetation within these contrasting landscapes.

We hypothesized that microbial and fungal community composition would vary in long-term lupine plots with gopher additions in the Pumice Plain as compared to those without historic gopher activity, and that we would find more mycorrhizal fungal mutualists in lupine plots containing historic gopher enclosures. We further hypothesized that the presence of animal dispersal vectors, such as the pocket gophers, will influence mycorrhizal guilds, community composition, and soil biogeochemical processes because of preferential foraging and other behaviors, including bioturbation, elimination (e.g., fecal deposits), and soil translocation.

## 2 Methods

### 2.1 Study site description

Prior to the May 18, 1980, eruption, the vegetation of our study sites on Mount St. Helens (46°16’N, 121°53’W) was primarily comprised of a Douglas fir forest interspersed with clearcuts (Allen et al., 1984, 1992). The area has predominately cool, wet winters and mild, dry summers, with heavy snowpack, often greater than 3 m. For this study, we focused on two locations: the Pumice Plain long-term lupine plot and Bear Meadow, a high-ashfall area in the path of the 1980 eruption.

The eruption itself created multiple zones or layers of disturbance and mortality, ranging from the crater, which is still active, to the Pumice Plain, the pyroclastic flow running out to Spirit Lake, down to ash deposition, from about 10 km to the northward extending as far north as Mount Rainier, and as far east as Lincoln, Nebraska. An overview is provided in **Figure 1**, **Supplementary Figure S1**, and in many publications (Allen et al., 1984; Dale et al., 2005; Allen et al., 2018). Additionally, Bear Meadow was a high-deposition area (**Supplementary Figure S1**). The Bear Meadow site consisted of two parts: a clearcut that became a meadow by 1980, and an adjacent old-growth Douglas fir forest.

### 2.2 Soil collection

On July 28 and 29, 2014, we collected soils from the Pumice Plain long-term lupine plots and from Bear Meadow, the high-ashfall area, which was in the path of the 1980 eruption. Three 9 cm diameter × 12 cm deep soil cores were collected and composited for each sample, for a total of 12 individual cores from the Pumice Plain (46°14’43.1”N 122°10’50.9”W), consisting of six composite samples from plots with historic Gopher enclosures and six composite samples from paired No Gopher control plots, also from the Pumice Plain. From Bear Meadow, 26 composite soil cores were collected across the clearcut (46°15’52.4”N 122°04’46.9”W) and old-growth (46°18’35.6”N 122°01’18.3”W) forests, with 11 samples from the clearcut and 15 from the old-growth. Surface litter and debris were removed before coring. Samples were immediately placed on dry ice for transportation to the laboratory at UC Riverside (Riverside, CA, USA). Soils were used for extraction and targeted amplicon sequencing, as well as to measure C and N concentrations.

### 2.3 Molecular analyses

We extracted microbial DNA from frozen soil (Pall, NY, USA). After collection in the field, samples were kept frozen in a -20°C freezer at the University of California, Riverside, until subsequent DNA extraction using a MoBio PowerSoil Power Lyzer DNA Isolation Kit (MO BIO Laboratories, Carlsbad, CA, USA; Qiagen Inc. Valencia, CA, USA), followed by cell lysis directly on a MoBio PowerLyzer tissue disrupter (Qiagen Inc., Valencia, CA, USA), as per the manufacturer’s instructions. Prior to tissue lysis, we weighed 0.25g of soil from each sample, and subsequently homogenized these aliquots, following a modified protocol from the PowerSoil PowerLyzer DNA Extraction Kit protocol (Protocols.io link; MO BIO Laboratories, Carlsbad, CA, USA).

Extracted DNA was further purified with a PEG and carboxylated magnetic bead solution, using a 1:1 ratio of sample-to-bead solution as described in Rohland and Reich (2012). Purified DNA extracts were quantified using a NanoDrop 2000/2000c UV-Vis spectrophotometer (Thermo Fisher Scientific, Wilmington, DE, USA) and concentrations normalized to 10 ng/μl.

Normalized and purified DNA was used as a template to amplify each of three different taxonomically informative loci by polymerase chain reaction (PCR). We used a tailed primer strategy for sequencing library construction to first amplify each target locus with a subsequent amplification to add molecular indexes and Illumina flowcell adapter sequences (Alvarado et al., 2018). Bacteria and Archaea were characterized according to the v4 region of the 16S rRNA gene using the 515F and 806R primers (Caporaso et al., 2010a). General fungal communities were characterized according to the Internal Transcribed Spacer 2 rRNA region, with primer sets ITS4-fun and 5.8S-fun (Taylor et al., 2016); finally, we used AMF-specific primers WANDA (Dumbrell et al., 2011) and AML2 (Lee et al., 2008). Individual sequencing libraries were quantified by PicoGreen (Thermo Fisher Scientific) fluorescence and pooled for sequencing.

Sequencing was carried out on a MiSeq Desktop Sequencer (Illumina Inc, San Diego CA) running in paired end 2×300 mode at the University of California, Riverside Institute for Integrative Genome Biology. Sequences were submitted to the National Center for Biotechnology Information Sequence Read Archive under BioProject PRJNA1124544. Total sequencing depths overall for 16S were 4097169 reads; for ITS2, read depth was 1728368; for SSU, depth was 1601559 reads.

### 2.4 Bioinformatics

The fungal sequences were demultiplexed in QIIME 1.9.1 (Caporaso et al., 2010b; Caporaso et al., 2012), and quality control measures were employed (Edgar and Flyvbjerg, 2015) in USEARCH. We characterized the percentage of reads passing through filters across different read lengths; we then determined the appropriate lengths to trim and still retain high-quality reads before per-base quality plummeted. After trimming, we ran trimmed sequences through FastQC to compare reports and to ensure that adapters were absent and per-base and per-sequence qualities had improved. Since there was at least a 12 base pair overlap, we used paired ends of both forward and reverse reads for both the V4 region of the 16S rRNA gene for bacteria and archaea and from the fungal ITS2 region to infer amplicon sequence variants (ASV) assignments from our sequences using the Divisive Amplicon Denoising Algorithm (DADA2; dada). ASVs were assigned based on 99% sequence identity (Prodan et al., 2020; Callahan et al., 2017).

#### 2.4.1 Arbuscular mycorrhizal fungal SSU

We filtered demultiplexed files from the SSU locus using multiple_split_libraries_fastq.py command in QIIME 1.9.1 with Q (Phred) scores of 20 (q = 19) as a quality control parameter; we did not allow any low-quality base calls (r = 0), and we only retained reads possessing 95% of initial sequence length following quality truncation (p = 0.95). We used VSEARCH (Rognes et al., 2016) in uchime_denovo mode to screen for chimeras. We used Swarm (Mahe et al., 2014) with a d4 resolution for picking operational taxonomic units (i.e., OTUs) for our SSU data. This d4 resolution for local clustering threshold collapsed sequences with fewer than or equal to 4 differences into a single representative OTU, as long as it passed our stringent quality filtering threshold of q20. We used BLAST to assign taxonomy, with an e-value of 0.001, via the MaarjAM (Opik et al., 2010) database. Prior to analysis, we truncated the reference databases to include only the region of interest, as well as to limit any spurious results. We used the metagenomeSeq package of Bioconductor (Ihaka and Gentleman, 1996) in R (R: Development core team, 2004) to normalize our OTUs using cumulative sum scaling (CSS-normalization) prior to further analyses (CSS normalization attempts to avoid biases associated with marker gene surveys, which could result from uneven sequencing depth). Read counts are rescaled against a quantile that was determined by assessing the count deviations for each sample, as compared to the distribution of counts across all of our other samples.

#### 2.4.2 Bacterial 16S and fungal ITS2

We separated our sequence files for the 16S and the ITS2 regions before the Illumina MiSeq data was processed interactively on the High Performance Computing Cluster at UC Riverside. We conducted further quality control (Kassambara, 2018) and trimming in DADA2 and used the filterAndTrim command to filter reads based on read quality, read length, number of unknown bases, expected errors, and matches to the PhiX genome, as per Freund (2023; 10.5281/zenodo.5794553). The region lengths vary for ITS2; therefore, truncating based solely on specific lengths may be unreliable. We used paired-end ITS2 reads when assigning taxonomic features to the ASVs via DADA2, as detailed in Freund (Lee, 2019) (2023; v1.0.1 10.5281/zenodo.8264886). The DADA2 algorithm as per Callahan et al. (2016) uses a parametric error model with both inference and estimation as it determines error rates per sample. We constructed our feature tables for both loci (i.e., ASV tables), removed chimeric sequences, and assigned taxonomy to our assigned ASVs using the Silva database and the Ribosomal Database Project for 16S, the UNITE database for fungal ITS2, and the MaarjAM data base for SSU.

To examine the AMF community’s (18S) responses within a fungal functional group framework, we assigned families of Glomeromycotina to AMF functional groups (rhizophilic, edaphophilic, and ancestral) using AMF resource allocation patterns defined in previous studies (Phillips et al., 2019; Weber et al., 2019). We did not include sequences assigned to the taxon *Geosiphon pyriformis*, as reads reportedly identified as *G. pyriformis* are not considered as rhizophilic, ancestral, or edaphophilic AMF. This functional group framework is based on studies demonstrating that AMF are host-specific and exhibit diverse resource allocation patterns. Arbuscular mycorrhizal fungal families with high allocation to extraradical hyphae, including Gigasporaceae and Diversisporaceae, are members of the guild “edaphophilic”; those with high allocation to root colonization (i.e., Glomeraceae, Claroideoglomeraceae, and Paraglomeraceae) are characterized as belonging to the “rhizophilic” guild; families that allocate lower AM fungal hyphae to either root colonization or soil foraging than the aforementioned guilds are known as “ancestral” AMF families, and include Ambisporaceae, Archaeosporaceae, and Acaulosporaceae. Previous studies show that fungal taxa from the rhizophilic AMF functional group are important for protecting their host plant from pathogen colonization, while edaphophilic AM fungi putatively improve plant nutrient uptake.

To examine fungal and bacterial microbiomes, as well as the relationship between these communities from Bear Meadow and the long-term lupine plot, sequences were processed with DADA2, with components modified from Quantitative Insights Into Microbial Ecology 2 (QIIME2; Bolyen et al., 2019). This approach was used to determine the relationship between bacterial microbiome communities and host diet, rearing, and sterility variables. We removed low-quality and chimeric sequences and computed core microbiomes in QIIME; we amplified sterile PCR-grade water, as a negative control, which was processed alongside the DNA samples. After samples were extracted, amplified, and sequenced, any OTUs or ASVs that were present in the negative controls were removed from downstream analyses.

To account for uneven sequencing depth samples’ bacterial and fungal alpha diversity was determined using rarefied ASV counts. For 16S, we used a rarefied ASV table to 17644 reads per sample; for ITS2 used a rarefied ASV table with 20069 reads per sample. Distance matrices were constructed using Bray-Curtis metrics through the “diversity()” function in the vegan package in R, using the Shannon-Wiener index. Species richness was calculated using raw ASV counts via the “specnumber()” function in the vegan package for R. Alpha diversity and species richness across treatment conditions and treatment sources were compared, respectively, using Wilcoxon tests, performed with the “compare_means” function in the “ggpubr” package for R, as well as t.test commands through the base R package. We used the “total” option in the “decostand()” function in the vegan package in R, upstream of compositional analyses, which relativizes varying ASV counts by dividing row total from each cell in the row, standardizing all of the counts to each other within each respective sample (Borcard et al., 2011; Legendre and Legendre, 2012).

To determine if fungal or bacterial composition differed among samples, Bray-Curtis distance matrices were used to compare community samples and to visualize and explain differences among microbial communities. We used nonmetric multidimensional scaling (NMDS) plots of the Bray Curtis distances and PERMANOVA analyses of microbial community data using the “adonis()” function in the vegan package of R. NMDS stress values (MDS; “metaMDS()” command, package vegan) were reported for fungi and bacteria. PERMANOVA was used to compare fungal bacterial community structures across all forest management, assisted migration, or control groups based on the ASV composition. To summarize broad-level taxonomic patterns, we parsed our count matrix by taxonomy to view the relative abundance of major taxa or microbial richness by categories; we used a 1.0% similarity threshold to explore the relationships among the relative abundances of abundant taxa. Percent N, percent C, and C:N ratios were used, along with Shannon diversity and species richness (**Supplementary Figure S2**) to further compare across treatment sources and conditions.

## 3 Results

### 3.1 Land use histories, soil chemistry, and microbial communities

We found that microbial communities varied among forestry practices (n=26) and between long-term lupine sites with and without gophers (n=12). These findings related to detected differences in carbon (C) or nitrogen (N) concentrations in soil or tephra samples across our study sites (**Supplementary Table 1**). Contrary to our first hypothesis, our data suggests that old-growth forests did not harbor greater microbial diversity or taxa richness than clearcut forests, which led us to reject that hypothesis. Our second hypothesis, that microbial and fungal community composition would vary across different forestry regimes, was supported and likewise correlated to higher C:N in old-growth, as compared to clearcut meadows in Bear Meadow forests. Although our methods did not allow us to characterize the successional trajectory, we were able to examine differences in multiple components of the soil and microbial community within a post-disturbance context as related to microbial rescue (Mueller et al., 2020; Shade, 2023) and land use histories.

### 3.2 Soil chemistry

Carbon (C) and nitrogen (N) levels varied across land use histories and gopher activity in the Pumice Plain. Samples recovered from lupine plots in the Pumice Plain had lower C (p = 0.02) and N (p = 0.04) concentrations than samples from the surrounding forests (**Supplementary Table 1**). Lupine plots without gophers were more depleted in C (p = 0.03), with lower C:N (p = 0.04), than was detected in lupine plots with historic gopher activity. Nitrogen concentrations in soils from lupine plots with gophers were higher than the percentage of N from soils from lupine plots without historic gopher activity (p = 0.046; at alpha value threshold, corresponding to the threshold between significant and *marginally* significant findings).

Soils from old-growth forests have higher concentrations of C (p = 0.01) and N (p = 0.01) than were found in historically clearcut forests; with C:N significantly lower in clearcut soils than in those from old-growth forests (p < 0.001).

### 3.3 Bacterial and archaeal taxa communities

Counterintuitively, bacterial and archaeal taxa richness (i.e., microbial richness) and alpha diversity (i.e., microbial diversity) in Bear meadow (n = 26) were higher in clearcut soils than in soils from old-growth forests (richness p < 0.001; diversity p < 0.001). Indeed, these microbial communities from clearcuts were more diverse and characterized by higher bacterial and archaeal taxa richness than those from remnant old-growth forests. Overall, microbial diversity was higher in the long-term lupine plots in the Pumice Plain (n = 12) than was found in the surrounding forests (p = 0.03), yet microbial taxa richness was nearly equivalent (i.e., marginally higher; p = 0.045) in lupine plots to the nearby forested locations.

Although microbial richness and diversity were equivalent in Pumice Plain plots with or without historical gopher activity (richness, p = 0.52; diversity, p = 0.22), Pumice Plain lupine plots that contained historical gophers harbored significantly greater microbial taxa richness (p = 0.03) and diversity (p < 0.001) than were found in the surrounding old-growth forests from Bear Meadow. Microbial diversity in Pumice Plain plots without historic gopher activity was equivalent to levels in clearcut forests (p = 0.014). Yet we found higher microbial diversity in these lupine plots than in old-growth forests (p=0.002). However, microbial richness in lupine plots with and without historical gopher activity was equivalent to richness detected in either old-growth (p=0.10) or clearcut forests (p=0.10). Additionally, we did not detect any clinal differences with respect to richness or diversity, as deeper layers below the eruption had similar microbial taxa richness (p = 0.32) and diversity (p = 0.86) as the top layers in the profile with these old-growth forests.

**Figure 2:**
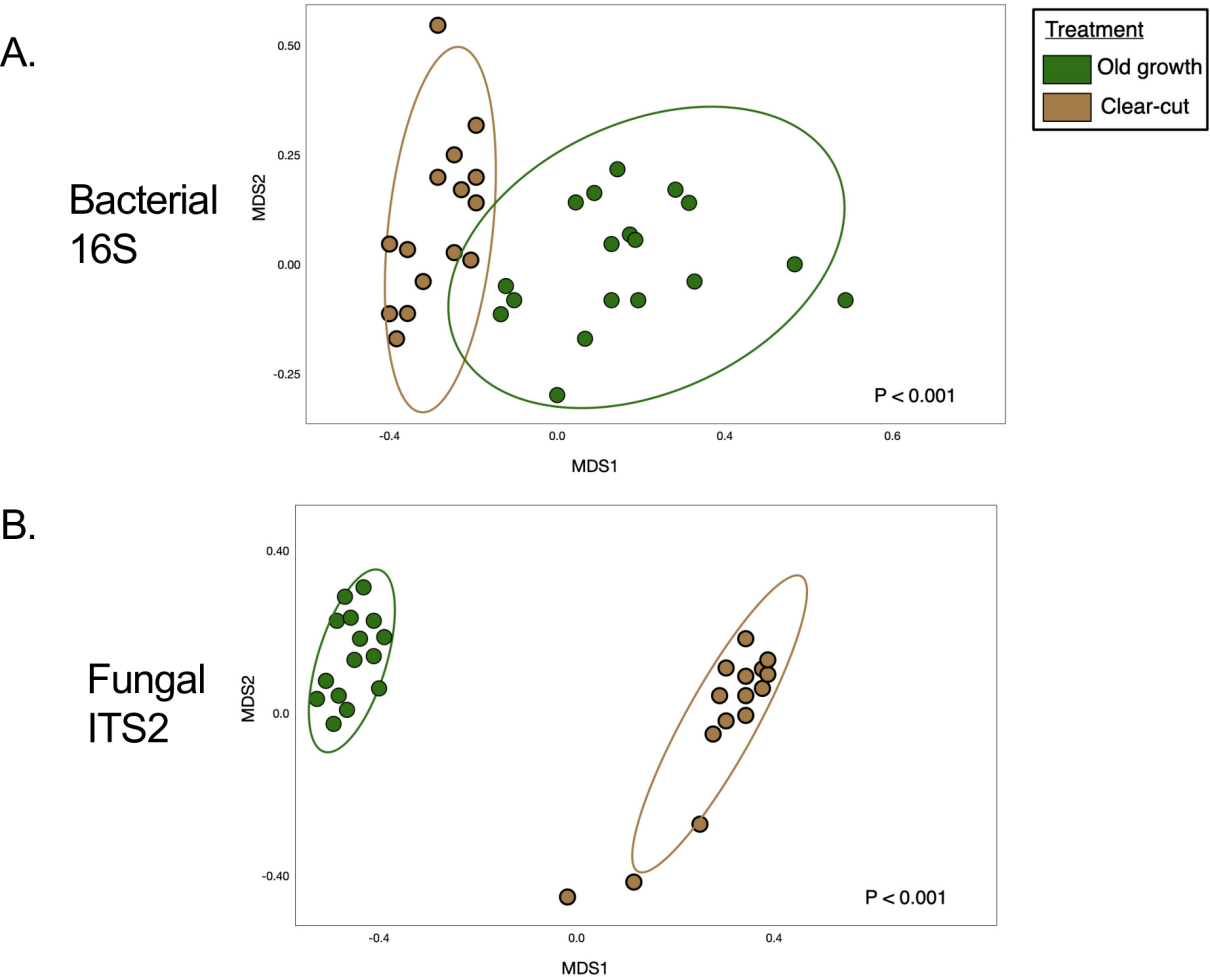
Microbial community composition shifts by land use histories in the forests surrounding Mount St. Helens. Non-metric multidimensional scaling (NMDS) based on bray-curtis distance matrices of bacterial **(A)**, and fungal **(B)**, communities revealed from targeted amplicon sequencing of 16S marker genes and fungal ITS2 region, illustrated by structural shifts by land use category in clearcut vs. old-growth forests **(A, B)** and lupine plots in the pumice plain. NMDS stress values (MDS; ‘metaMDS()’ command, package vegan) were 0.1272 for bacteria and 0.1031 for fungi. PERMANOVA analyses illustrated by structural shifts in the ordination by land use category in clearcut vs. old-growth forests **(A, B)**.

The composition of microbial communities in the lupine plots differed significantly from that of the surrounding forests (p < 0.001), with old-growth microbial communities differing from the clearcut forests (p = 0.002; **Figure 2**). PERMANOVA was used to compare bacterial community structures across all forest management (MDS stress value 0.132), assisted migration (MDS stress value 0.164), or control groups based on the ASV composition), as visualized via NMDS. Taxa from Acidomicrobiia and Planctomycetes were more common in old-growth than in clearcut forests, with taxa from Gammaproteobacteria more commonly found in clearcut forests than were detected in old-growth forests within this system.

PERMANOVA analyses revealed that microbial and fungal community composition correlated significantly with soil C:N (p < 0.001), as well as the percent C (p < 0.001) and N (p < 0.001) of soil samples. Additionally, microbial community composition varied by layer in the profile or regolith, as microbial community structure from the top layer of soil in the Bear Meadow old-growth forests differed significantly from those in the layers below (p = 0.02) the previous eruption. Additionally, the composition of microbial communities from the lupine plots with gophers differed significantly from the composition of those without gophers (p = 0.01).

### 3.4 Fungal communities

Fungal taxa richness (i.e., fungal richness) and alpha diversity (i.e., fungal diversity) in clearcut soils were equivalent to soils from old-growth forests (richness p = 0.06; diversity p = 0.05; **Supplementary Figure S2**). Overall, fungal diversity was higher in the lupine plots in the Pumice Plain than was found in the surrounding forests (p = 0.01); yet no differences were detected in fungal taxa richness in lupine plots, as compared to samples from the surrounding forested locations.

Fungal diversity was significantly greater in lupine plots with gophers than in clearcut forests (p < 0.02); however, fungal richness was similar across these same conditions (p = 0.10). Fungal richness was equivalent across lupine plots with gophers and the surrounding old-growth forest (richness, p = 0.38). Although lupine plots without gophers also harbored more diverse fungal communities than were found in old-growth forests (p < 0.001), fungal taxa richness was equivalent in old-growth forests as compared to those in the lupine plots without historic gopher activity.

Fungal composition varied significantly by forest management strategies, with clearcut forests characterized by different fungal communities than those found in the surrounding old-growth forests (p < 0.001; **Figure 1**; MDS stress value 0.127). Likewise, we detected structural differences in fungal communities in gopher plots as compared to “no-gopher” lupine plots (p = 0.01; assisted migration MDS stress value 0.150).

Fungal community composition likewise varied significantly by the percentage of N (p < 0.001), C (p < 0.001), and the C:N (p < 0.001) of soil or tephra samples. Moreover, fungal community composition in the old-growth forests of Bear Meadows differed with depth, with significantly different fungal communities found in the top layer of the old growth, as compared to the layers below (p = 0.03).

**Figure 3:**
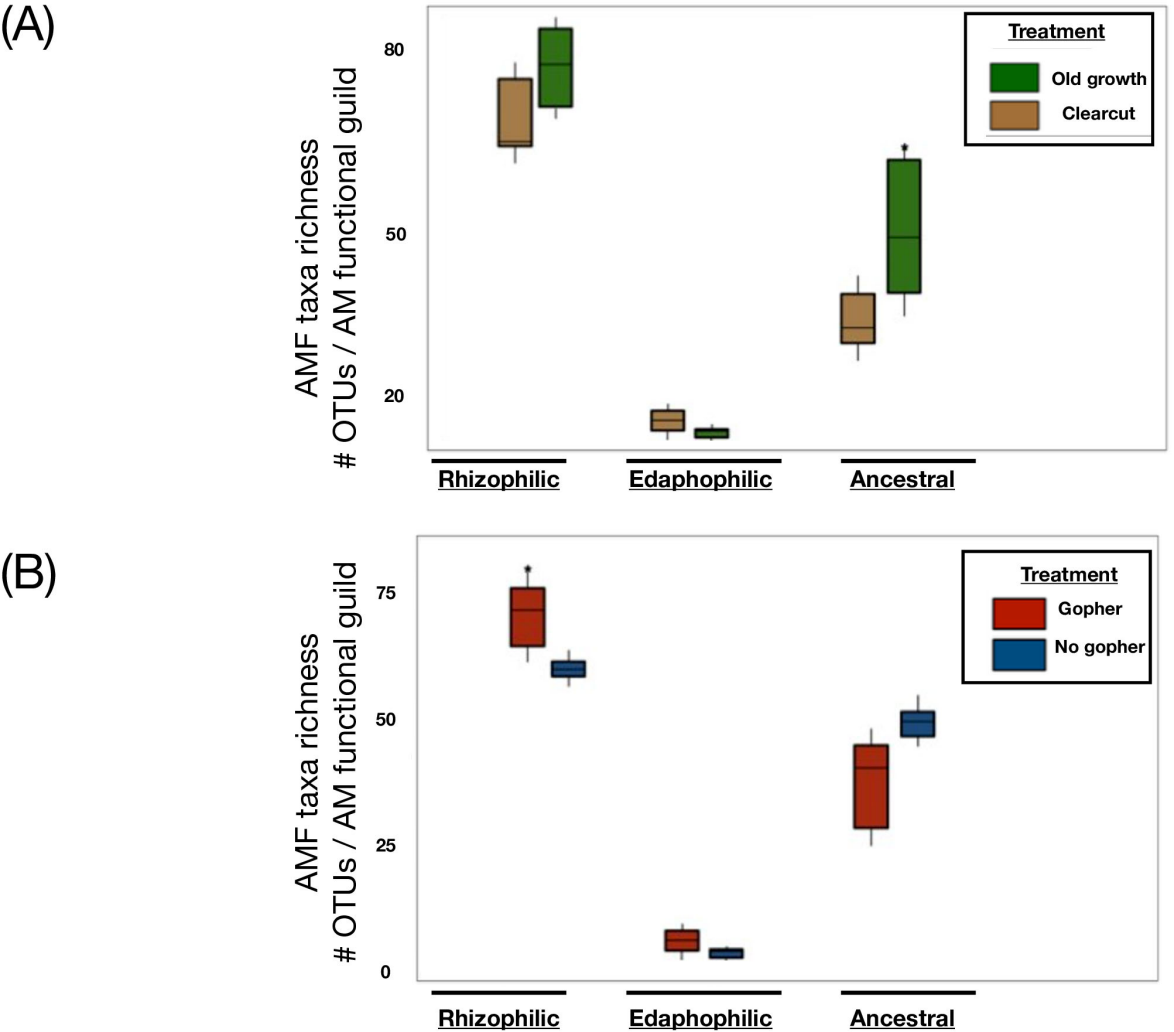
Arbuscular mycorrhizal fungal guilds shifted by dispersal vectors and land use histories in the forests surrounding Mount St. Helens. Arbuscular mycorrhizal composition shifts by historical land use and dispersal vectors in the forests surrounding Mount St. Helens. Richness of AMF guilds refers to OTU’s (operational taxonomic unit, equivalent to molecular species concept) in fungal families represented by functional guilds (as per Weber et al., 2019) in these different forest conditions **(A)** or plots differing by gopher presence or absence **(B)**. Taxa and guild assignments revealed from targeted amplicon sequencing of 18S region and universal eukaryote Wanda primer set to assign families of Glomeromycotina to AMF functional guilds. Far left groupings in each panel have been assigned to rhizophilic AM families, middle groups were assigned to edaphophilic AMF, and the far right group in each panel encompass the taxonomic richness calculated from within ancestral AMF families. Taxa richness within these guilds were compared within AM functional group in plots of old growth vs. clearcut forests **(A)**, while the **(B)** depicts plots comparing AM taxa richness within guilds in either ‘Gopher’ or ‘No gopher’ plots.

A majority of the fungi detected in this system belonged to the Ascomycota, with a greater proportion of the old-growth forest represented by Basidiomycota, and specifically Agaricomycetes, than were present in the clearcut forests. Mushroom-forming fungal genera, such as *Inocybe*, *Cortinarius*, and *Craterellus* sp., and ectomycorrhizal fungi, including *Piloderma* and *Wilcoxina* sp., were more commonly found in old-growth forests. Ascomycete fungi, such as the plant disease-forming *Paraphoma* and *Paraphaeosphaeria*, as well as plant-supporting *Mortierella* (Mucoromycotina) and basidiomycetous yeasts and jelly fungi, including *Solicoccozyma*, more often in the soil from clearcut forests. More taxa from the Glomeromycotina were found in lupine plots with gophers than were found in locations without historic gopher activity. These arbuscular mycorrhizal fungi from Glomeromycotina varied according to functional guilds, with greater abundance of “ancestral” (as per Powell et al., 2009; Weber et al., 2019) mycorrhizal fungi in old-growth forests than clearcut forests. Ancestral taxa include those from Archaeosporacacae, Ambisporacacae, Pacisporacacae (as per Varela-Cervero et al., 2015), and Acaulosporacacae fungal families. Additionally, in long-term lupine plots with gophers from the Pumice Plain, we detected a greater richness of “rhizophilic” mycorrhizal fungi (i.e., Families: Glomeraceae, Claroideoglomeraceae, and Paraglomeraceae; Hart and Reader, 2002; Varela-Cervero et al., 2016a, 2016; **Figure 3**). With greater richness of rhizophilic AM taxa found in the lupine plots with gophers, we had partial support for our hypotheses related to more mycorrhizal fungi—in this case, specifically taxa from the rhizophilic guild—being detected in these historic gopher-containing plots within the Pumice Plain.

## 4 Discussion

We found evidence supporting long-term differences in microbial communities from the legacy effects of land use and assisted migration (i.e., gopher dispersal vectors) following the 1980 eruption of Mount St. Helens. The transformative effects of volcanic eruptions on soil-forming factors and biotic communities in surrounding ecosystems beget the trajectory dynamics of post-disturbance recovery, especially as important plant, animal, and microbial taxa return to establish on previously sterile pumice and tephra. The structure and legacy of forests, including pre-eruption aboveground biomass and volcanic eruptions, likely influenced soil microbial communities (Chen et al., 2023). Our study showed that microbial and fungal communities from the high-ashfall area in the path of the 1980 eruption remained vertically stratified with regolith in the profile, even decades after the 1980 blast; selective environmental pressure originating from multiple eruption events over time may have impacted the resultant soil microbiomes.

Biotic factors influenced long-term lupine plots, as gophers translocated both plant and microbial propagules around the Pumice Plain. As gophers naturally bioturbated, defecated, and mixed soil and tephra layers, these actions amended depauperate locations following the pyroclastic flow. In our study, we detected the presence of more types of root-associated mycorrhizal taxa (i.e., rhizophilic) in gopher plots than we found in long-term lupine plots without historic gopher activity. These mutualistic, rhizophilic AMF taxa may have promoted plant establishment within hostile terrain while also protecting plant roots from threats such as competition from root-associated pathogens (Borowicz, 2001; Powell et al., 2009; Weber et al., 2019). Plots with historic gopher activity harbored more diverse bacterial and fungal communities than the surrounding old-growth forests. We also found more diverse fungal communities in these long-term lupine gopher plots than in forests that were historically clearcut, prior to the 1980 eruption, nearby at Bear Meadow. Over 40 years since the blast, legacy or priority effects may have led to differences in fungal and microbial community composition across contrasting forestry regimes, or in pairwise treatments from within the long-term lupine plot at the Pumice Plain, upslope from Spirit Lake (**Supplementary Figure S3**). Indeed, the most dramatic compositional differences were detected in fungal communities from the previously clearcut forest, as compared to the old-growth forest surrounding Bear Meadow.

Ecologists are increasingly finding evidence that land use changes and forest management strategies could affect the long-term structure, functioning, and resilience of managed ecosystems. When lava cools, successional (Ni et al., 2023) forces initiate novel ecosystems and assemblages; invading organisms and immigrating propagules may be dispersed via aeolian processes. As subsets of biotic propagules survive within a patchy mosaic throughout the landscape (Allen et al., 1992), bacteria are some of the first organisms to succeed in primary succession, followed by other microorganisms and ruderal taxa that can either modify or tolerate these harsh conditions within the ashfall and blast zone (Abdulla, 2009). Some extremotolerant taxa could be some of the first pioneers in these novel environments (Ghosh et al., 2010). Some microorganisms exude proteins, such as those that break down harmful oxygen species or molecules that promote osmoregulation or extreme temperature tolerance, or find refugia in forming biofilms that protect bacterial cells physically, mechanically, or chemically; these microbial products ostensibly support their survival in toxic soils disturbed by volcanic eruptions (Memoli et al., 2018; Chen et al., 2023). As extreme conditions alter biodiversity and competitive interactions, or enhance abiotic or environmental filtering, this may both limit invasions by exotic species and preclude extant taxa from persisting or proliferating in harsh environments. These factors likely exert selective pressure on the local community, which may hinder the establishment of immigrating taxa (Williamson and Harrison, 2002) and drive community assembly processes.

We found that bacterial community composition varied, based on pre-eruption forest management strategies or through altering propagule pressure via biotic introductions. While some members of biotic communities may have survived the blast, deep in profile, others—albeit smaller taxa—likely arrived via wet deposition as exogenous inputs from entrained ash or air currents (Maltz et al., 2022). Although bacteria vary in their tolerance of stressful environmental conditions, those that proliferated following the pyroclastic flow likely were equipped with a particular combination of traits that allowed them to persist in low-nutrient environments, such as traits related to carbohydrate synthesis and storage or the ability to form biofilms or spores (Brewer et al., 2019).

As the cataclysmic As the cataclysmic Mount St. Helens eruption destroyed thousands of hectares of forest and montane habitat, it blanketed the surrounding adjacent old-growth *Pseudotsuga menziesii* (i.e., Douglas fir) forest and clearcut in Bear Meadow with nearly 20 cm of ash. Remarkably, deeply packed snow protected remnant soil organisms that survived the eruption. Other taxa survived the blast by a combination of protection via rotten logs, soil, erosional forces, and topographic features (Andersen, 1982; Andersen and MacMahon, 1985; Allen et al., 2005). Animals, such as gophers, ants, mountain-dwelling pika (*Ochotona daurica*), and other taxa, served as vectors of dispersal. These ecosystem engineers amid the volcanic rubble were wont to move deep organic soil from below the ashfall to the surface, effectively mixing decayed organic matter with the ash and depositing nutrients via physical bioturbation.

Gophers translocated and deposited diverse soil microbiomes, seeds, and mycorrhizal spores from their fecal matter (Allen et al., 2005) into recipient ecosystems. Microbial communities were likely seeded by gophers and other remnant, immigrant, or ruderal taxa serving as dispersal vectors. This could have accelerated physical and biochemical weathering of the Andisol volcanic parent material with unconsolidated Andic Haplocryods soils and material (Web Soil Survey, Staff). Organic acids and residues from biotic introductions may also have enhanced the production of stable soil aggregates. Although measuring soil aggregation was beyond the scope of this study, we demonstrate that such interactions were also important in directly affecting microbial community composition and habitat, as well as the C:N of soil environments.

Pumice Plain plots with historic gopher activity had more C and a higher C:N than those locations without gopher interventions or activity. Additionally, N and C concentration and C:N were higher in old-growth forests than in the Bear Meadow clearcut, which was related to fungal and microbial community composition. In forest soils, soil microbes may play key roles in biotic immobilization of NH_4_^+^ to soil organic matter (Brookes et al., 1985), as microbially-derived organic N. Halvorson et al. (as per Dale et al., 2005) showed that prairie lupine at the pyroclastic flow site maintained higher nitrogenase activity, which corresponded with diurnal patterns of plant photosynthesis and heightened N-fixation along the successional trajectory in the Pumice Plain. Likewise, nitrate leaching may be related to microbial community activity, as C:N may influence the release of essential minerals, such as zinc and phosphorus, as well as N mineralization, immobilization, and nitrification rates (van Veen et al., 1984; Bengtsson et al., 2003). Our study shows that both fungal and microbial community composition were related to C:N and percent N within these recovering forest soils. This suggests that these environmental microbiomes not only responded to environmental cues, but their role in biogeochemical cycling also may have affected their surrounding environments, such as through enhancing plant acquisition, bioavailability, or the lability of limited nutrients and minerals within this system.

Although the previously clearcut forests in Bear Meadow harbored more bacterial taxa than nearby old-growth forests, fungal taxa richness and diversity were unchanged across Bear Meadow regardless of forest management methods. Yet we detected more diverse bacterial, archaeal, and fungal communities in the lupine plots within the Pumice Plain than we did at Bear Meadow. Microbial biodiversity is often linked to enhanced ecosystem function and to both ecological and evolutionary drivers of terrestrial ecosystem response to environmental changes (Bardgett and van der Putten, 2014). Rather than diversity or taxa richness being the overarching indicator of forest recovery, our work detected varying degrees of microbial and fungal sensitivity to forest management, introductions of historical gophers, and soil C and N concentrations. Although we did not have comparable molecular evidence characterizing composition pre-eruption, our findings suggest that the compositional differences across extant conditions may be long-lasting, or may not attenuate, even at decadal time scales.

This study showed that old-growth forests harbored more types of root-associated mutualistic fungi from the ancestral AMF guild than were found in the clearcut forest from Bear Meadow. Arbuscular mycorrhizal fungi are affected by soil moisture (Augé, 2001) and nutrient availability (Egerton-Warburton and Allen, 2000). Although we still have more to learn about the functional role of this cryptic AM group, which neither allocates excessive fungal hyphae within plant roots (i.e., intraradical) nor extends substantially into the interstitial places within the soil (i.e., extrametrical or extraradical) hyphae (Hart and Reader, 2002; Powell et al., 2009; Varela-Cervero et al., 2016a; Weber et al., 2019), we may aggregate AM communities based on trait-based approaches that assign AMF to functional groups based on physiological traits. These “ancestral AMF” may tolerate low soil moisture or nutrient levels while promoting plant performance in stressful or harsh environments, such as the Pumice Plain. AM life-history strategies and morphological features such as extraradical hyphae production may be advantageous for nutrient cycling, while genomic attributes, including genes for metabolic maintenance and survival, such as trehalose synthesis, may confer benefits in resource-limited soils (Brewer et al., 2019). Given their unclear allocation preferences, additional research into the functional variation within ancestral AM families may elucidate mechanisms for ecological specialty and inform our predictive framework for understanding which plant species are sensitive to benefits conferred by ancestral AM. Moreover, environmental parameters, such as water or N deficiencies, may also prime ancestral AM support of their host plants or fungal biogeochemical cycling within novel ecosystems. As the frequency and severity of natural disasters increase, and as water or nutrient availability becomes limited, ancestral AM associations may be increasingly important in understanding successional dynamics, such as after major volcanic eruptions.

Multi-trophic interactions, such as AMF spore dispersal by small mammals, may ameliorate the impacts of severe disturbance and promote succession on the Pumice Plain of Mount St. Helens. Microbial propagules dispersed throughout the Pumice Plain likely pave the way for ecological succession by promoting soil aggregation, thereby preventing erosion and topsoil sloughing. As pumice material eroded downslope, it exposed new soils, and erosional rills and gullies served as “hotspots” for resprouting residual plants (Allen et al., 2005, in Dale et al., 2005). In this recovering system, spores also may have been deposited and established in the soil profile, along with plants and microbial residues (e.g., organic acids, microbial exudates, and sticky glycoproteins), which accumulate and aggregate soils, thereby influencing soil structure in these sensitive ecosystems.

Land use patterns may alter topsoil retention and soil health, which could play an important role in ecosystem resilience. Microorganisms illustrate environmental preferences, which could contribute toward our capacity to predict their responses to environmental change (Delgado-Baquierizo et al., 2018). Moreover, as ecosystems are altered by eruption, losses of dominant species could open niches to transient taxa. However, Grime’s (1998) mass-ratio hypothesis suggests that disproportionate impacts on key soil processes, soil C, and biogeochemical cycling could be driven by primary production rates and the functional diversity of dominant plants. Likewise, belowground successional pathways and long-term retention or recovery of ecological communities could be affected differentially by forest management methods, such as whether conventional tree removal or sustainable forestry methods were employed in the decades preceding the 1980 eruption. Different trends in community assembly may be explained by land use or forest management methods, as well as whether stochastic or deterministic processes dominate successional dynamics.

Findings from our study could help us evaluate whether the rules that govern macro-community assembly in natural systems also apply to microbial communities following volcanic eruptions (Pérez-Hernández and Gavilán, 2021). Our work examined fungal and bacterial communities in the historic blast zone of Mount St. Helens. It highlighted structural differences in microbial groups exhibiting particular life history strategies or traits that may underscore their own survival or their host retention. Despite their small size, microbes may be key to understanding how communities respond to severe disturbances in novel ecosystems. Our investigation of these natural processes is germane for addressing contemporary climate crises and ecosystems recovering from anthropogenic activities. Likewise, we can learn much from examining the strategies and structure of macro- and microorganisms that establish and proliferate in resource-limited, harsh, or inhospitable environments, aided by dispersal vectors. As successional processes, biotic legacies, and priority effects interact to create novel biotic communities in unlikely places following eruptions, microbial processes influence their proximal environments in creating microsite conditions, which may be conducive to biotic survival, owing to spatial constraints. Findings from our study show that, after disturbance, microbial reestablishment may have been partitioned by historic forest management practices and augmented by assisted migrations (as per Shade et al., 2023; microbiome rescue), which could have long-term impacts on successional dynamics, microbial communities, and host-symbiont interactions.

## Data Availability

The original contributions presented in the study are publicly available. This data can be found here: NCBI SRA, accession PRJNA1124544, http://www.ncbi.nlm.nih.gov/bioproject/1124544.

## References

[B1] Abdulla H. (2009). Bioweathering and biotransformation of granitic rock minerals by actinomycetes. Microbial Ecol. 58, 753–761. doi: 10.1007/s00248-009-9549-1 19590809

[B2] Allen M. F. Crisafulli C. Friese C. F. Jeakins S. L. (1992). Re-formation of mycorrhizal symbioses on Mount St Helens 1980–1990: interactions of rodents and mycorrhizal fungi. Mycological Res. 96, 447–453. doi: 10.1016/S0953-7562(09)81089-7

[B3] Allen M. F. Crisafulli C. M. Morris S. J. Egerton-Warburton L. M. MacMahon J. A. Trappe J. M. (2005). Mycorrhizae and Mount St. Helens: story of a symbiosis. Ecological responses to the 1980 eruption of Mount St. Helens. New York, NY: Springer. 221–231.

[B4] Allen M. F. MacMahon J. A. (1988). Direct VA mycorrhizal inoculation of colonizing plants by pocket gophers (*Thomomys talpoides*) on Mount St. Helens. Mycologia 80, 754–756. doi: 10.1080/00275514.1988.12025615

[B5] Allen M. F. MacMahon J. A. Andersen D. C. (1984). Reestablishment of Endogonaceae on Mount St. Helens: survival of residuals. Mycologia 76, 1031–1038. doi: 10.1080/00275514.1984.12023947

[B6] Allen M. F. O’Neill M. R. Crisafulli C. M. MacMahon J. A. (2018). “Succession and mycorrhizae on mount st. Helens,” in Crisafulli C. Dale V. (eds) Ecological responses at Mount St. Helens: revisited 35 years after the 1980 eruption. New York, NY: Springer. 199–215.

[B7] Alvarado P. Teixeira M. D. M. Andrews L. Fernandez A. Santander G. Doyle A.. (2018). Detection of Coccidioides posadasii from xerophytic environments in Venezuela reveals risk of naturally acquired coccidioidomycosis infections. Emerging Microbes infections 7, 1–13. doi: 10.1038/s41426-018-0049-6 29593263 PMC5874253

[B8] Andersen D. C. (1982). Observations on Thomomys talpoides in the region affected by the eruption of Mount St. Helens. J. Mammalogy 63, 652–655. doi: 10.2307/1380271

[B9] Andersen D. C. MacMahon J. A. (1985). The effects of catastrophic ecosystem disturbance: the residual mammals at Mount St. Helens. J. Mammalogy 66, 581–589. doi: 10.2307/1380942

[B10] Augé R. M. (2001). Water relations, drought and vesicular-arbuscular mycorrhizal symbiosis. Mycorrhiza 11, 3–42. doi: 10.1007/s005720100097

[B11] Banwart S. A. Nikolaidis N. P. Zhu Y. G. Peacock C. L. Sparks D. L. (2019). Soil functions: connecting earth's critical zone. Annu. Rev. Earth Planetary Sci. 47, 333–359. doi: 10.1146/annurev-earth-063016-020544

[B12] Bardgett R. D. van der Putten W. H. (2014). Belowground biodiversity and ecosystem functioning. Nature 515, 505–511. doi: 10.1038/nature13855 25428498

[B13] Baross J. A. Dahm C. N. Ward A. K. Lilley M. D. Sedell J. R. (1982). Initial microbiological response in lakes to the Mt St Helens eruption. Nature 296, 49–52. doi: 10.1038/296049a0

[B14] Bengtsson G. Bengtson P. Månsson K. F. (2003). Gross nitrogen mineralization-, immobilization-, and nitrification rates as a function of soil C/N ratio and microbial activity. Soil Biol. Biochem. 35, 143–154. doi: 10.1016/S0038-0717(02)00248-1

[B15] Bolyen E. Rideout J. R. Dillon M. R. Bokulich N. A. Abnet C. C. Al-Ghalith G. A.. (2019). Reproducible, interactive, scalable and extensible microbiome data science using QIIME 2. Nat. Biotechnol. 37, 852–857. doi: 10.1038/s41587-019-0209-9 31341288 PMC7015180

[B16] Borcard D. Gillet F. Legendre P. (2011). Numerical ecology with R Vol. 2 (New York: springer), 688).

[B17] Borowicz V. A. (2001). Do arbuscular mycorrhizal fungi alter plant–pathogen relations? Ecology 82, 3057–3068. doi: 10.1890/0012-9658(2001)082[3057:DAMFAP]2.0.CO;2

[B18] Bowd E. J. Banks S. C. Bissett A. May T. W. Lindenmayer D. B. (2022). Disturbance alters the forest soil microbiome. Mol. Ecol. 31, 419–447. doi: 10.1111/mec.16242 34687569

[B19] Brewer T. E. Aronson E. L. Arogyaswamy K. Billings S. A. Botthoff J. K. Campbell A. N.. (2019). Ecological and genomic attributes of novel bacterial taxa that thrive in subsurface soil horizons. MBio 10, 10–1128. doi: 10.1128/mBio.01318-19 PMC6775450 31575762

[B20] Brookes P. C. Landman A. Pruden G. Jenkinson D. S. (1985). Chloroform fumigation and the release of soil nitrogen: a rapid direct extraction method to measure microbial biomass nitrogen in soil. Soil Biol. Biochem. 17, 837–842. doi: 10.1016/0038-0717(85)90144-0

[B21] Callahan B. J. McMurdie P. J. Holmes S. P. (2017). Exact sequence variants should replace operational taxonomic units in marker-gene data analysis. ISME J. 11, 2639–2643. doi: 10.1038/ismej.2017.119 28731476 PMC5702726

[B22] Callahan B. J. McMurdie P. J. Rosen M. J. Han A. W. Johnson A. J. A. Holmes S. P. (2016). DADA2: High-resolution sample inference from Illumina amplicon data. Nat. Methods 13, 581–583. doi: 10.1038/nmeth.3869 27214047 PMC4927377

[B23] Caporaso J. G. Kuczynski J. Stombaugh J. Bittinger K. Bushman F. D. Costello E. K.. (2010a). “QIIME allows analysis of high-throughput community sequencing data,” in Nature methods, vol. 7 (5), 335–336. doi: 10.1038/nmeth.f.303 20383131 PMC3156573

[B24] Caporaso J. G. Lauber C. L. Walters W. A. Berg-Lyons D. Huntley J. Fierer N.. (2012). Ultra-high-throughput microbial community analysis on the Illumina HiSeq and MiSeq platforms. ISME J. 6, 1621–1624. doi: 10.1038/ismej.2012.8 22402401 PMC3400413

[B25] Caporaso J. G. Lauber C. L. Walters W. A. Berg-Lyons D. Lozupone C. A. Turnbaugh P. J.. (2010b). Global patterns of 16S rRNA diversity at a depth of millions of sequences per sample. Proc. Nat. Acade. Sci. 108, 4516–4522. doi: 10.1073/pnas.1000080107 PMC3063599 20534432

[B26] Chen J. Xiao Q. Xu D. Li Z. Chao L. Li X.. (2023). Soil microbial community composition and co-occurrence network responses to mild and severe disturbances in volcanic areas. Sci. Total Environ. 901, 165889. doi: 10.1016/j.scitotenv.2023.165889 37524180

[B27] Coba de la Pena T. Fedorova E. Pueyo J. J. Lucas M. M. (2018). The symbiosome: legume and rhizobia co-evolution toward a nitrogen-fixing organelle? Front. Plant Sci. 8, p.2229. 10.3389/fpls.2017.02229 PMC5786577 29403508

[B28] Dale V. H. Swanson F. J. Crisafulli C. M. (2005). Ecological responses to the 1980 eruptions of mount st (New York: Helens. Springer-Verlag).

[B29] Delgado-Baquerizo M. Oliverio A. M. Brewer T. E. Benavent-González A. Eldridge D. J. Bardgett R. D.. (2018). A global atlas of the dominant bacteria found in soil. Science 359, 320–325. doi: 10.1126/science.aap9516 29348236

[B30] Dove N. C. Keeton W. S. (2015). Structural complexity enhancement increases fungal species richness in northern hardwood forests. Fungal Ecol. 13, 181–192. doi: 10.1016/j.funeco.2014.09.009

[B31] Dumbrell A. J. Ashton P. D. Aziz N. Feng G. Nelson M. Dytham C.. (2011). Distinct seasonal assemblages of arbuscular mycorrhizal fungi revealed by massively parallel pyrosequencing. New Phytol. 190, 794–804. doi: 10.1111/j.1469-8137.2010.03636.x 21294738

[B32] Edgar R. C. Flyvbjerg H. (2015). Error filtering, pair assembly and error correction for next-generation sequencing reads. Bioinformatics 31, 3476–3482. doi: 10.1093/bioinformatics/btv401 26139637

[B33] Egerton-Warburton L. M. Allen E. B. (2000). Shifts in arbuscular mycorrhizal communities along an anthropogenic nitrogen deposition gradient. Ecol. Appl. 10, 484–496. doi: 10.1890/1051-0761(2000)010[0484:SIAMCA]2.0.CO;2

[B34] Findley R. (1981). Mount St. Helens, Mountain with a death wish. Natl. geographic 159, 3–33.

[B35] Franklin J. F. (2005). Reconfiguring disturbance, succession, and forest management: The Science of Mount St. Helens. Eds. Dale V. H. Swanson F. J. Crisafulli C. M. (Springer-Verlag, New York: Ecological Responses to the 1980 Eruptions of Mount St. Helens), V–VII.

[B36] Freund H. L. (2023). doi: 10.5281/zenodo.8264886

[B37] Fruchter J. S. Robertson D. E. Evans J. C. Olsen K. B. Lepel E. A. Laul J. C.. (1980). Mount St. Helens ash from the 18 May 1980 eruption: chemical, physical, mineralogical, and biological properties. Science 209, 1116–1125. doi: 10.1126/science.209.4461.1116 17841472

[B38] Ghosh S. Osman S. Vaishampayan P. Venkateswaran K. (2010). Recurrent isolation of extremotolerant bacteria from the clean room where Phoenix spacecraft components were assembled. Astrobiology 10, 325–335. doi: 10.1089/ast.2009.0396 20446872

[B39] Grime J. P. (1998). Benefits of plant diversity to ecosystems: immediate, filter and founder effects. J. Ecol. 86, 902–910. doi: 10.1046/j.1365-2745.1998.00306.x

[B40] Halvorson J. J. Smith J. L. Kennedy A. C. (2005). “Lupine effects on soil development and function during early primary succession at Mount St. Helens,” in Ecological responses to the 1980 eruption of Mount St. Helens (Springer New York, New York, NY), 243–254).

[B41] Hart M. M. Reader R. J. (2002). Taxonomic basis for variation in the colonization strategy of arbuscular mycorrhizal fungi. New Phytol. 153, 335–344. doi: 10.1046/j.0028-646X.2001.00312.x

[B42] Hernandez Garcia M. Calabi-Floody M. Conrad R. Dumont M. (2020). Analysis of the microbial communities in soils of different ages following volcanic eruptions: Microbial communities in volcanic soils. Pedosphere 30, 126–134. doi: 10.1016/S1002-0160(19)60823-4

[B43] Ihaka R. Gentleman R. (1996). R: A Language for Data Analysis and Graphics}. Journal of Computational and Graphical Statistics. (5)3:299–314.

[B44] Jackson A. C. Jorna J. Chaston J. M. Adams B. J. (2022). Glacial legacies: microbial communities of Antarctic refugia. Biology 11, p.1440. doi: 10.3390/biology11101440 PMC9598129 36290344

[B45] Kassambara A. (2018). ggpubr: 'ggplot2'-based publication ready plots. R package version, Vol. 2.

[B46] Lee J. Lee S. Young J. P. W. (2008). Improved PCR primers for the detection and identification of arbuscular mycorrhizal fungi. FEMS Microbiol. Ecol. 65, 339–349. doi: 10.1111/fem.2008.65.issue-2 18631176

[B47] Lee M. D. (2019). Happy Belly Bioinformatics: an open-source resource dedicated to helping biologists utilize bioinformatics. J. Open Source Educ. 4, p.53. doi: 10.21105/jose.00053

[B48] Legendre P. Legendre L. (2012). Numerical ecology (Vol. 24). Elsevier. New York, NY.

[B49] Logan W. B. (2007). Dirt: The ecstatic skin of the earth (New York, NY: WW Norton & Company).

[B50] MacMahon J. A. Warner N. (1984). “Dispersal of mycorrhizal fungi: processes and agents,” in VA mycorrhizae and reclamation of arid and semiarid lands (University of Wyoming Press, Laramie), 28–41.

[B51] Mahé F. Rognes T. Quince C. de Vargas C. Dunthorn M. (2014). Swarm: robust and fast clustering method for amplicon-based studies. PeerJ 2, e593. doi: 10.7717/peerj.593 25276506 PMC4178461

[B52] Maltz M. R. Carey C. J. Freund H. L. Botthoff J. K. Hart S. C. Stajich J. E.. (2022). Landscape topography and regional drought alters dust microbiomes in the sierra nevada of california. Front. Microbiol. 13, 856454. doi: 10.3389/fmicb.2022.856454 35836417 PMC9274194

[B53] McCook L. J. (1994). Understanding ecological community succession: Causal models and theories, a review. Vegetatio 110, 115–147. doi: 10.1007/BF00033394

[B54] Memoli V. Eymar E. García-Delgado C. Esposito F. Panico S. C. De Marco A.. (2018). Soil element fractions affect phytotoxicity, microbial biomass and activity in volcanic areas. Sci. total Environ. 636, 1099–1108. doi: 10.1016/j.scitotenv.2018.04.327 29913572

[B55] Mueller E. A. Wisnoski N. I. Peralta A. L. Lennon J. T. (2020). Microbial rescue effects: how microbiomes can save hosts from extinction. Funct. Ecol. 34, 2055–2064. doi: 10.1111/1365-2435.13493

[B56] Ni G. Lappan R. Hernández M. Santini T. Tomkins A. G. Greening C. (2023). Functional basis of primary succession: Traits of the pioneer microbes. Environ. Microbiol. 25, p.171. doi: 10.1111/1462-2920.16266 PMC10098604 36309943

[B57] Öpik M. Vanatoa A. Vanatoa E. Moora M. Davison J. Kalwij J. M.. (2010). The online database MaarjAM reveals global and ecosystemic distribution patterns in arbuscular mycorrhizal fungi (Glomeromycota). New Phytol. 188, 223–241. 20561207 10.1111/j.1469-8137.2010.03334.x

[B58] Perez-Hernandez J. Gavilan R. G. (2021). Impacts of land-use changes on vegetation and ecosystem functioning: old-field secondary succession. Plants 10, 990. doi: 10.3390/plants10050990 34065656 PMC8156868

[B59] Phillips J. D. Lorz C. (2008). Origins and implications of soil layering. Earth-Science Rev. 89, 144–155. doi: 10.1016/j.earscirev.2008.04.003

[B60] Phillips M. L. Weber S. E. Andrews L. V. Aronson E. L. Allen M. F. Allen E. B. (2019). Fungal community assembly in soils and roots under plant invasion and nitrogen deposition. Fungal Ecol. 40, 107–117. doi: 10.1016/j.funeco.2019.01.002

[B61] Picone N. Hogendoorn C. Cremers G. Poghosyan L. Pol A. van Alen T. A.. (2020). Geothermal gases shape the microbial community of the volcanic soil of Pantelleria, Italy. MSystems 5, 10–1128. doi: 10.1128/mSystems.00517-20 PMC7646524 33144309

[B62] Powell J. R. Parrent J. L. Hart M. M. Klironomos J. N. Rillig M. C. Maherali H. (2009). Phylogenetic trait conservatism and the evolution of functional trade-offs in arbuscular mycorrhizal fungi. Proceedings of the Royal Society B. Biol. Sci. 276, 4237–4245. 10.1098/rspb.2009.1015 PMC2821337 19740877

[B63] Prodan A. Tremaroli V. Brolin H. Zwinderman A. H. Nieuwdorp M. Levin E. (2020). Comparing bioinformatic pipelines for microbial 16S rRNA amplicon sequencing. PloS One 15, e0227434. doi: 10.1371/journal.pone.0227434 31945086 PMC6964864

[B64] Pulsford S. A. Lindenmayer D. B. Driscoll D. A. (2016). A succession of theories: purging redundancy from disturbance theory. Biol. Rev. 91, 148–167. doi: 10.1111/brv.12163 25428521

[B65] Quince C. Walker A. W. Simpson J. T. Loman N. J. Segata N. (2017). Shotgun metagenomics, from sampling to analysis. Nat. Biotechnol. 35, 833–844. doi: 10.1038/nbt.3935 28898207

[B66] R: Development core team. (2004). R: A language and enviroment for statistical computing. R foundation for statistical computing, Vienna, Austria. http://www.r-project.org

[B67] Reichman O. J. Seabloom E. W. (2002). The role of pocket gophers as subterranean ecosystem engineers. Trends Ecol. Evolution. 17, 44–49. doi: 10.1016/S0169-5347(01)02329-1

[B68] Rognes T. Flouri T. Nichols B. Quince C. Mahé F. (2016). VSEARCH: a versatile open source tool for metagenomics. PeerJ 4, e258. doi: 10.7717/peerj.2584 PMC5075697 27781170

[B69] Rohland N. Reich D. (2012). Cost-effective, high-throughput DNA sequencing libraries for multiplexed target capture. Genome Res. 22, 939–946. doi: 10.1101/gr.128124.111 22267522 PMC3337438

[B70] Shade A. (2023). Microbiome rescue: directing resilience of environmental microbial communities. Curr. Opin. Microbiol. 72, p.102263. doi: 10.1016/j.mib.2022.102263 36657335

[B71] Stothers R. B. (1984). The great Tambora eruption in 1815 and its aftermath. Science 224, 1191–1198. doi: 10.1126/science.224.4654.1191 17819476

[B72] Taylor D. L. Walters W. A. Lennon N. J. Bochicchio J. Krohn A. Caporaso J. G.. (2016). Accurate estimation of fungal diversity and abundance through improved lineage-specific primers optimized for Illumina amplicon sequencing. Appl. Environ. Microbiol. 82, 7217–7226. doi: 10.1128/AEM.02576-16 27736792 PMC5118932

[B73] van Veen J. A. Ladd J. N. Frissel M. J. (1984). Modeling C and N turnover through the microbial biomass in soil. Plant Soil 76, 257–274. doi: 10.1007/BF02205585

[B74] Varela-Cervero S. López-García Á. Barea J. M. Azcón-Aguilar C. (2016a). Differences in the composition of arbuscular mycorrhizal fungal communities promoted by different propagule forms from a Mediterranean shrubland. Mycorrhiza 26, 489–496. doi: 10.1007/s00572-016-0687-2 26883142

[B75] Varela-Cervero S. López-García Á. Barea J. M. Azcón-Aguilar C. (2016b). Spring to autumn changes in the arbuscular mycorrhizal fungal community composition in the different propagule types associated to a Mediterranean shrubland. Plant Soil 408, 107–120. doi: 10.1007/s11104-016-2912-3

[B76] Varela-Cervero S. Vasar M. Davison J. Barea J. M. Öpik M. Azcón-Aguilar C. (2015). The composition of arbuscular mycorrhizal fungal communities differs among the roots, spores and extraradical mycelia associated with five Mediterranean plant species. Environ. Microbiol. 17, 2882–2895. doi: 10.1111/1462-2920.12810 25677957

[B77] Vitousek P. M. (2004). Nutrient cycling and limitation: Hawai'i as a model system ( Princeton New Jersey: Princeton University Press).

[B78] Vuong H. B. Thrall P. H. Barrett L. G. (2017). Host species and environmental variation can influence rhizobial community composition. J. Ecol. 105, 540–548. doi: 10.1111/1365-2745.12687

[B79] Wagg C. Bender S. F. Widmer F. van der Heijden M. G. (2014). Soil biodiversity and soil community composition determine ecosystem multifunctionality. Proc. Natl. Acad. Sci. 111, 5266–5270. doi: 10.1073/pnas.1320054111 24639507 PMC3986181

[B80] Weber S. E. Diez J. M. Andrews L. V. Goulden M. L. Aronson E. L. Allen M. F. (2019). Responses of arbuscular mycorrhizal fungi to multiple coinciding global change drivers. Fungal Ecol. 40, 62–71. doi: 10.1016/j.funeco.2018.11.008

[B81] Williamson J. Harrison S. (2002). Biotic and abiotic limits to the spread of exotic revegetation species. Ecol. Appl. 12, 40–51. doi: 10.1890/1051-0761(2002)012[0040:BAALTT]2.0.CO;2

[B82] Yokobe T. Hyodo F. Tokuchi N. (2020). Volcanic deposits affect soil nitrogen dynamics and fungal–bacterial dominance in temperate forests. Soil Biol. Biochem. 150, 108011. doi: 10.1016/j.soilbio.2020.108011

[B83] Zimmerman N. Izard J. Klatt C. Zhou J. Aronson E. (2014). The unseen world: environmental microbial sequencing and identification methods for ecologists. Front. Ecol. Environ. 12, 224–231. doi: 10.1890/130055

