# Supplementary material for: Microbial community structure in recovering forests of Mount St. Helens
Source: Front Microbiomes. 2024 Nov 4;3:1399416. doi: 10.3389/frmbi.2024.1399416 (PMC12993564; doi:10.3389/frmbi.2024.1399416)
Supplement: Supplementary file 1 [file DataSheet1.pdf]

Supplementary Material

1 Supplementary Data

Maltz, Mia (Forthcoming 2024). Microbial Feature Tables and Nutrient Data regarding fungal and bacterial community structure in recovering forests of Mount St. Helens [Dataset]. Dryad.

The datasets generated and analyzed for this study can be found in the data dryad site, accessible at: [https://doi.org/10.5061/dryad.ksn02v7c6].

2 Supplementary Figures and Tables

2.1 List of Supplementary Material

2.1.1 Supplementary Figure 1 Photographs of the Bear Meadow forested site.

2.1.2 Supplementary Figure 2 Microbial richness showing categorical groupings of richness by plot type.

2.1.3 Supplementary Figure 3 Map illustration of Mount St. Helens and the surrounding area

2.1.4 **Supplementary Table 1** Nutrient status of soils from Mount St. Helens from Bear Meadow contrasting 1980 and 2000, ash and soil, and 2015, old growth, clearcut, and from 2015 in the Pumice Plain, contrasting long-term Lupine plots with gophers and without historic gopher activity

2.2 Supplementary Figures

2.2.1 Supplementary Figure 1 Photographs of the Bear Meadow forested site.

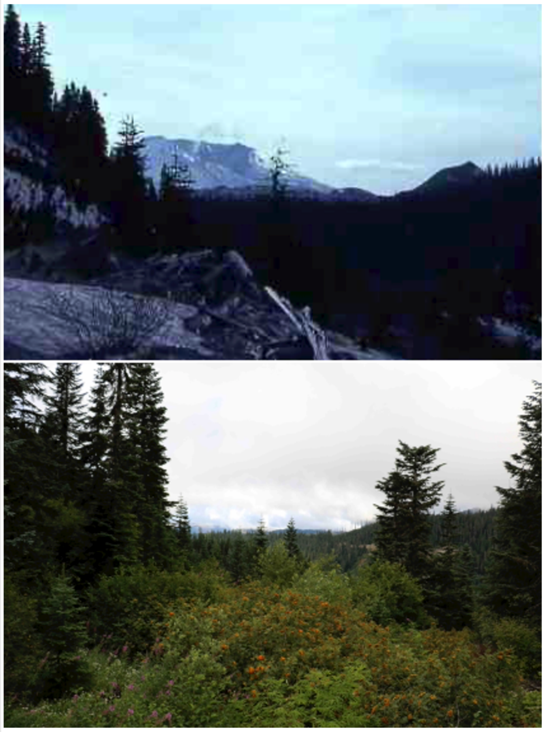

Supplementary Figure 1 Photographs of the Bear Meadow forested site. An overview of the Bear Meadow site from 1981, with the clearcut-meadow (A) shown above an photograph of the old-growth Douglas fir forest in Bear Meadow from 2015 (B).

2.2.2 Supplementary Figure 2 Microbial richness showing categorical groupings of richness by plot type.

2.2.3 **Supplementary Figure 2 Microbial richness showing categorical groupings of richness by plot type.** Microbial richness from molecular analyses of targeting amplicon sequencing of bacterial 16S rRNA gene (panel A) and fungal ITS2 (panel B) amplicons showing taxa richness varies by plot type, comparing either clearcut vs. old-growth forests or long-term lupine plots with either gophers present or absent in the recovering Pumice Plain.

.

A. **B.**

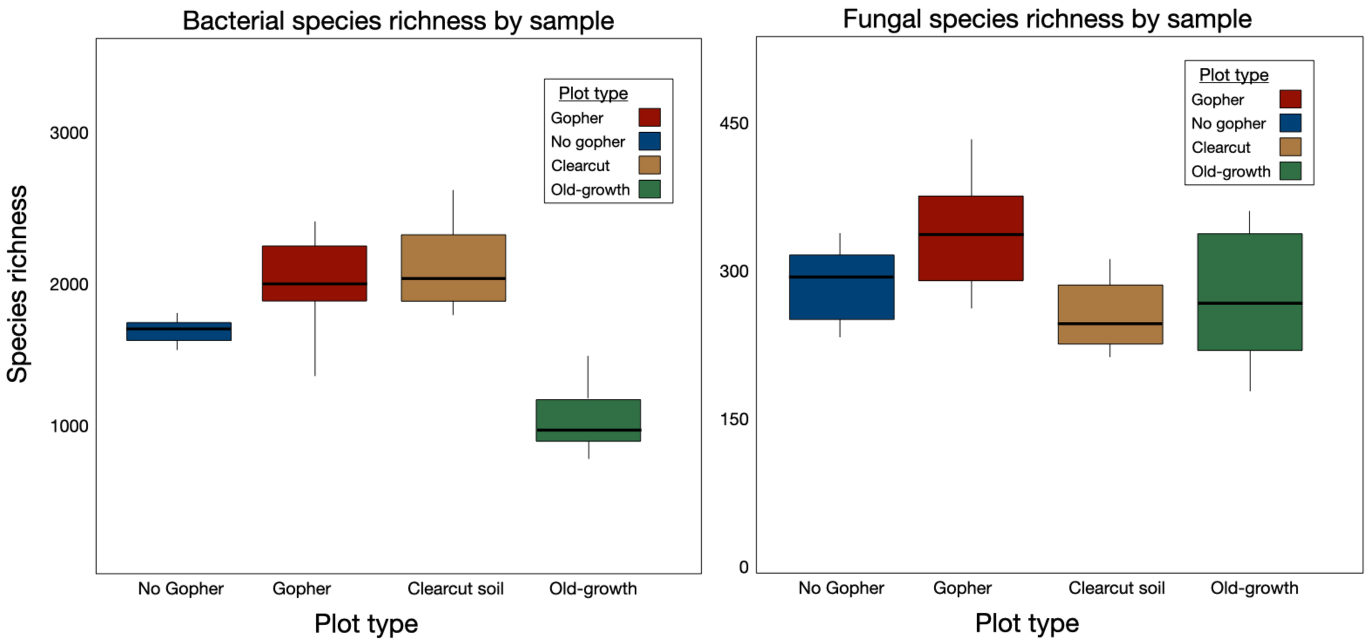

2.2.4 Supplementary Figure 3 Map illustration of Mount St. Helens and the surrounding area.

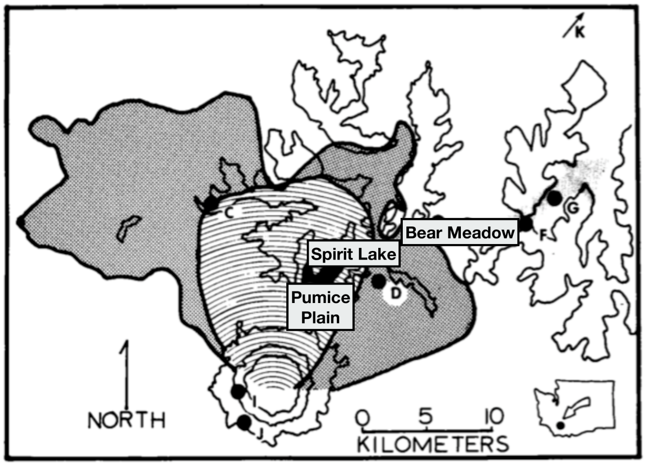

Supplementary Figure 3 Map illustration of Mount St. Helens and the surrounding area. Locations of sampling sites of Mount St. Helens and the areas in close proximity, with the Pumice Plain, Spirit Lake, and Bear Meadow labeled on the map. Inner directed blast zone and blowdown area shown here, engulfed the Pumice Plain near Spirit Lake and abutted the Bear Meadow clearcut and old growth forests.

2.3 Supplementary Table

2.3.1 Supplementary Table 1 Nutrient status of soils from Mount St. Helens from Bear Meadow, contrasting 1980 and 2000, ash and soil, and 2015, old growth, clearcut, and from 2015 in the Pumice Plain, contrasting long-term Lupine plots with gophers and without historic gopher activity. From 1980 and 2000, Carbon values shown are organic C; with 1980 data collected by MacMahon and Warner (1984) and 2000 data collected by Morris and Allen, as per Dale et al. 2005; in 2015 total C values are reported as means ± SE.

**Parameter**	**Value**	**1980**		**2000**		**2015**		**2015**	
		Tephra	Soil	Tephra	Soil	Old Growth	Clearcut	Gopher	No Gopher
**Carbon**	g/kg	2.2	39.6	1.46	17.5	122.39 ±38	0.09 ±0.21	9.54 ±01.18	4.33 ±0.48
**Nitrogen, total**	mg/kg	NA	NA	0.01	390	2860 ±69	60.0 ±30	1070 ±130	560 ±0.40
